# Surface-Engineered Phytochemical Nanomedicines for Postmenopausal Osteoporosis: Skeletal Targeting, Imaging-Supported Evaluation, and Therapeutic Performance

**DOI:** 10.3390/pharmaceutics18091163

**Published:** 2026-09-16

**Authors:** Hongwei Gao, Shaorong Li, Han Wu, Weijian Wang, Jianshi Song, Jiaqi Li, Xiuqi Shan, Wei Zhang

**Affiliations:** Department of Spinal Surgery, The Third Hospital of Hebei Medical University, Shijiazhuang 050001, China

**Keywords:** postmenopausal osteoporosis, phytochemicals, surface-engineered nanomedicines, skeletal targeting, mineral affinity, imaging-supported evaluation, ovariectomized model, controlled release

## Abstract

Postmenopausal osteoporosis (PMOP) is a metabolic bone disease driven by estrogen deficiency and a major contributor to fragility fractures, yet long-term use of current antiresorptive and anabolic agents remains constrained by tolerability and adherence. Plant-derived bioactive compounds—including flavonoids, isoflavones, curcuminoids, and stilbenes—show bone-protective activity in ovariectomized (OVX) models but share common delivery barriers: poor aqueous solubility, low oral bioavailability, rapid metabolic clearance, and limited skeletal targeting. Conventional nanonization improves systemic pharmacokinetics but cannot by itself address the spatial heterogeneity of skeletal target sites. This review focuses on surface-engineered phytochemical nanomedicines, in which mineral-affinity, cell-affinity, and pathology-responsive modules are introduced at the carrier interface to enhance bone-associated distribution, local retention, and microenvironment-responsive release. Organized around nanocarrier platforms, it summarizes cross-platform surface/interfacial engineering strategies and discusses how whole-body, ex vivo, and bone-section imaging can be combined with quantitative exposure assessment, micro-computed tomography (micro-CT), bone turnover markers, and histology to characterize carrier- or probe-associated distribution, spatial localization, and therapeutic performance, respectively. It then considers translational issues, including formulation reproducibility, ligand-density effects, model relevance, and long-term safety. The review aims to provide a framework for surface-engineered phytochemical strategies and a reference approach for multimodal, imaging-based evaluation of therapeutic outcomes.

## 1. Introduction

Postmenopausal osteoporosis (PMOP) and estrogen-deficiency osteoporosis (EDO) are common metabolic bone diseases in older women, characterized by reduced bone mass and deterioration of bone microarchitecture that gradually increase the risks of fragility fracture, subsequent fracture, functional impairment, and mortality [1]. The period after a first fracture is an important window for secondary prevention, but guideline-concordant treatment rates and long-term adherence remain inadequate among individuals at high fracture risk [2,3,4,5]. Antiresorptive agents, including bisphosphonates, selective estrogen receptor modulators, and denosumab, have established efficacy, but their long-term use remains complicated by safety, tolerability, adherence, and the need to maintain treatment continuity after discontinuation [4,5,6]. Therefore, adjunctive strategies that complement existing drugs and are suitable for long-term use remain of practical interest.

Phytochemicals offer diverse therapeutic options for mitigating PMOP/EDO. Flavonoids, isoflavones, and related polyphenols can counter estrogen-deficiency-associated bone loss by regulating osteogenic differentiation, osteoclast activity, oxidative stress, inflammatory responses, and the gut microbiota–bone metabolism axis [7,8,9,10,11]. Representative compounds include icariin, naringin, quercetin, puerarin, curcumin, and resveratrol, which have been shown to improve bone mass, trabecular architecture, and bone turnover markers in the ovariectomized (OVX) model—a standard preclinical model in which surgical removal of the endogenous estrogen source reproduces PMOP/EDO-associated bone loss [12,13,14,15,16].

However, for most of these compounds, absorption, distribution, metabolism, and excretion (ADME) barriers jointly shape efficacy and safety and are central to their pharmaceutical developability [11,17,18,19,20]. Phytochemicals are constrained by poor aqueous solubility, low oral bioavailability, extensive first-pass metabolism, limited stability, and rapid clearance, which together make their actual exposure in bone tissue difficult to predict [7,8].

**Figure 1:**
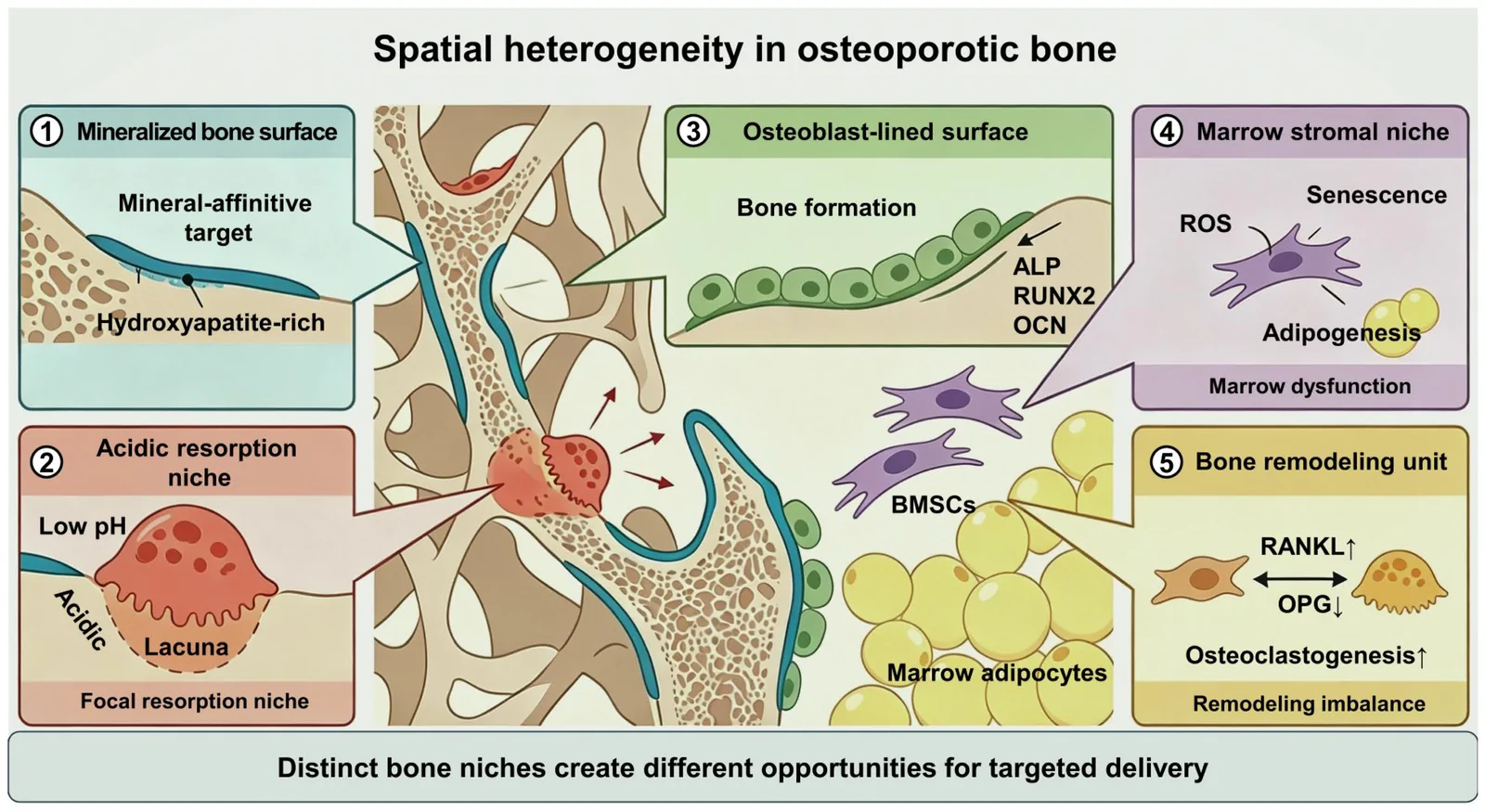
Spatial heterogeneity in osteoporotic bone creates distinct mineral, resorption, osteogenic, marrow-stromal, and remodeling niches for targeted delivery.

Conventional nanotechnologies, including liposomes, solid lipid nanoparticles (SLNs), polymeric nanoparticles, micelles, and nanocrystals, can improve the dispersibility, stability, release behavior, and systemic exposure of poorly soluble compounds [21,22,23]. However, nanonization mainly improves systemic pharmacokinetics, whereas the therapeutic target regions in PMOP/EDO are spatially heterogeneous, spanning mineralized bone surfaces, active bone-remodeling units, osteoclast-associated acidic microdomains, and bone marrow cellular niches. Formulations that merely increase plasma concentrations or systemic exposure still cannot achieve effective local concentrations at skeletal target sites, nor can they provide release that matches the pathological microenvironment (Figure 1).

This need for spatial specificity has driven skeletal delivery research from simple nanonization toward surface engineering. By modifying the carrier interface, three categories of strategies can be introduced: mineral-affinity modules, including bisphosphonate/phosphonate groups, acidic peptides, and tetracycline-based bone-affinity structures; cell-affinity modules, including cell-targeting peptides and biomimetic membranes; and pathology-responsive modules, including pH-, ROS-, and enzyme-responsive units. These strategies are intended to enhance binding to bone mineral, local retention, and pathology-responsive release [24,25,26,27,28]. Surface-engineered phytochemical nanomedicines can therefore not only improve solubility and systemic exposure but also enhance bone-associated distribution and local pharmacological effects.

Recent reviews have discussed, from various perspectives, the bone-protective actions of phytochemicals, functionalized or bone-targeted nanoparticles in osteoporosis, and broader bone-targeted nanodrug and nanodelivery strategies. The review by Wu et al. systematically surveyed a range of nanomaterials and bone-targeting strategies from the broader field of bone-related diseases and bone-targeted delivery [29], whereas other recent reviews have focused, respectively, on the bone-protective actions of phytochemicals, functionalized nanoparticles in osteoporosis, or bone-targeted nanodelivery [7,28,30,31]. These works provide an important foundation for the field. Accordingly, this review does not attempt to reproduce a general catalogue of bone-targeted nanodelivery spanning all bone diseases, carrier materials, and targeting ligands, nor is its primary aim to re-summarize the broad bone-protective pharmacology of phytochemicals. By contrast, this review narrows its scope to phytochemical nanodelivery in PMOP/EDO and related OVX models, and is organized around three interrelated dimensions. First, at the level of the delivered cargo, phytochemicals are taken as the primary focus—with flavonoids and isoflavones representing the most extensively studied class—and emphasis is placed on the delivery barriers shared by these compounds, including insufficient aqueous solubility, limited oral bioavailability, first-pass metabolism, inadequate stability, and rapid clearance. Second, at the level of engineering design, nanocarrier structure and surface/interfacial engineering are treated as interrelated but relatively distinct design dimensions, allowing mineral-affinity, cell-affinity, and pathology-responsive strategies to be compared across lipid, polymeric/polyphosphazene, organic-inorganic hybrid, biomimetic, and carrier-free/supramolecular platforms. Third, at the level of delivery evaluation, this review focuses on which conclusions different experimental readouts can support: in vitro hydroxyapatite (HAp) binding primarily reflects mineral affinity; fluorescence and near-infrared (NIR) imaging primarily reflect carrier- or probe-associated distribution; bone-section imaging can provide tissue- or cell-level spatial localization; and liquid chromatography-tandem mass spectrometry (LC-MS/MS) tissue-concentration measurement together with tissue-specific quantitative pharmacokinetics can more directly support the assessment of actual cargo exposure. Building on this analytical framework, this review first discusses representative phytochemical cargoes and their shared delivery barriers, then analyzes different nanoplatforms along the two dimensions of carrier structure and surface/interfacial engineering, and further examines delivery-evaluation approaches—including imaging, tissue localization, and quantitative exposure and their relationship to therapeutic outcomes. Finally, it discusses formulation reproducibility, model relevance, long-term safety, and translational positioning.

### Literature Search and Review Scope

**Figure 2:**
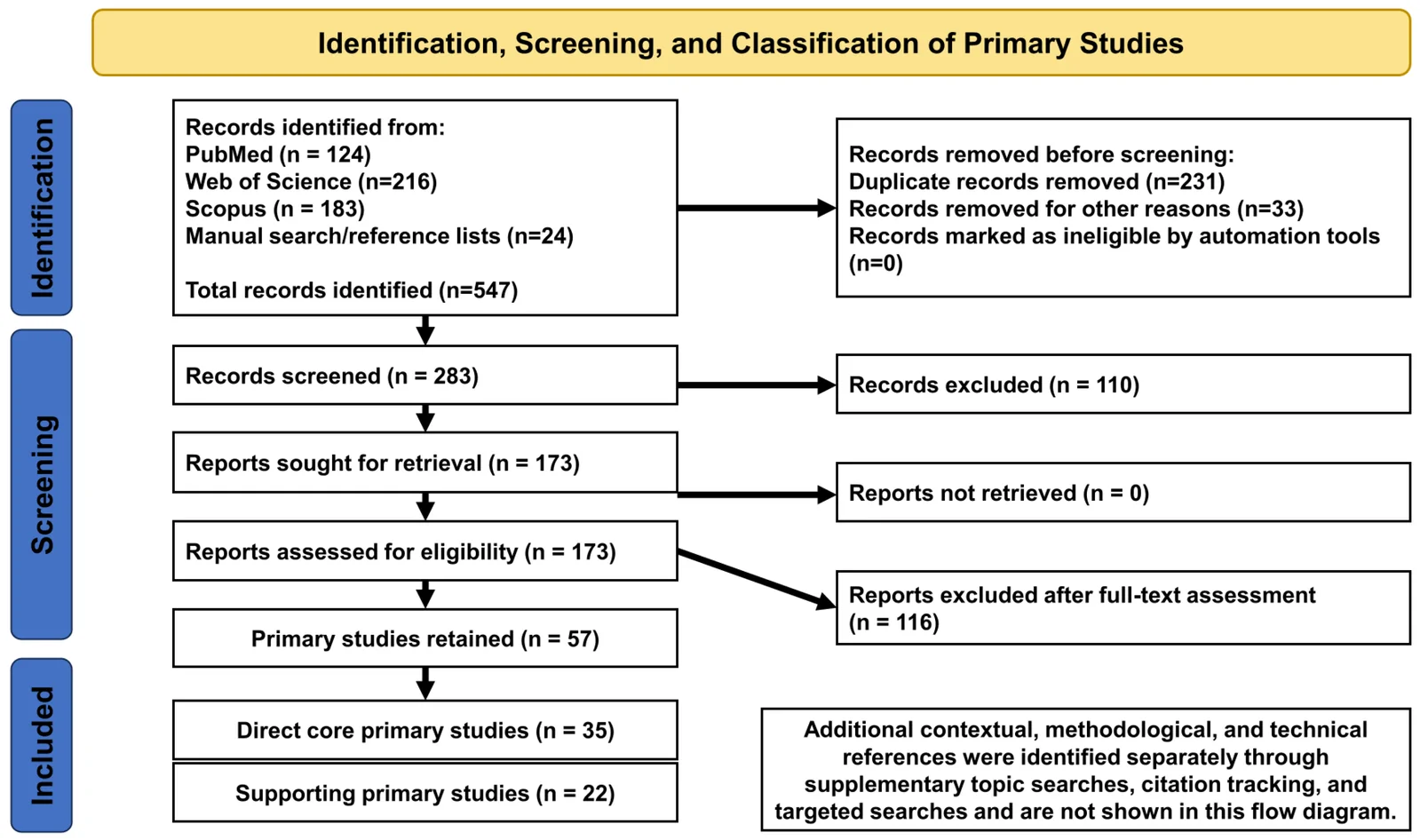
The literature identification, screening and classification process of this structured narrative review.

This article is a structured narrative review. Predefined search strategies and selection criteria were applied to identify primary studies relevant to the main analytical questions of this review. Screening was performed by a single researcher, without protocol registration or formal risk-of-bias assessment. Figure 2 uses a PRISMA-style flow diagram to display the identification, screening, and classification of primary studies, so as to improve the transparency of the search process.

PubMed, Web of Science, and Scopus were searched from inception to 10 June 2026, supplemented by reference-list screening of key articles; only English-language publications were included. The representative search strategy was: ((“postmenopausal osteoporosis” OR “estrogen-deficiency osteoporosis” OR ovariectom* OR OVX) AND (phytochemical* OR flavonoid* OR isoflavone* OR curcumin OR resveratrol OR icariin OR naringin) AND (nanomedicine* OR nanoparticle* OR “surface engineering” OR “bone targeting” OR “hydroxyapatite binding”) AND (biodistribution OR imaging OR pharmacokinetic*)). The search syntax was adapted to the rules of each database.

Searching and screening identified 547 records, of which 57 primary studies relevant to the scope of this review were finally retained and further classified according to their relevance to the main analytical questions. Of these, 35 were categorized as directly core primary studies, comprising mainly those evaluating phytochemical nanoformulations, surface/interfacial engineering, bone-associated distribution or localization, quantitative cargo exposure, and therapeutic performance in OVX, PMOP, EDO, or directly related osteoporosis models. These 35 studies constitute the principal basis for the delivery-evidence appraisal, cross-platform comparison, and structured descriptive evidence synthesis in this review. A further 22 were categorized as supporting primary studies, used mainly to substantiate the pharmacological basis of phytochemicals, delivery barriers, pharmacokinetics, bioavailability, and related evidence in adjacent models, but were not incorporated into the descriptive synthesis centered on the incremental effect of surface/interfacial engineering. Studies confined to bone-defect or bone-regeneration models with no direct relevance to systemic PMOP/EDO delivery, as well as reviews, editorials, and duplicate reports, were not included in the directly core evidence set. Other background, methodological, and technical references were obtained through supplementary topic searches, reference-list tracking, and targeted searches, and were used to support discussion of mechanisms, imaging methods, safety, regulatory aspects, clinical positioning, adjacent technologies, and comparison with previous reviews; these references are not counted among the 57 primary studies above. The identification, screening, and classification of primary studies are shown in Figure 2.

## 2. Phytochemicals in Nanomedicines for Estrogen-Deficiency Osteoporosis

**Figure 3:**
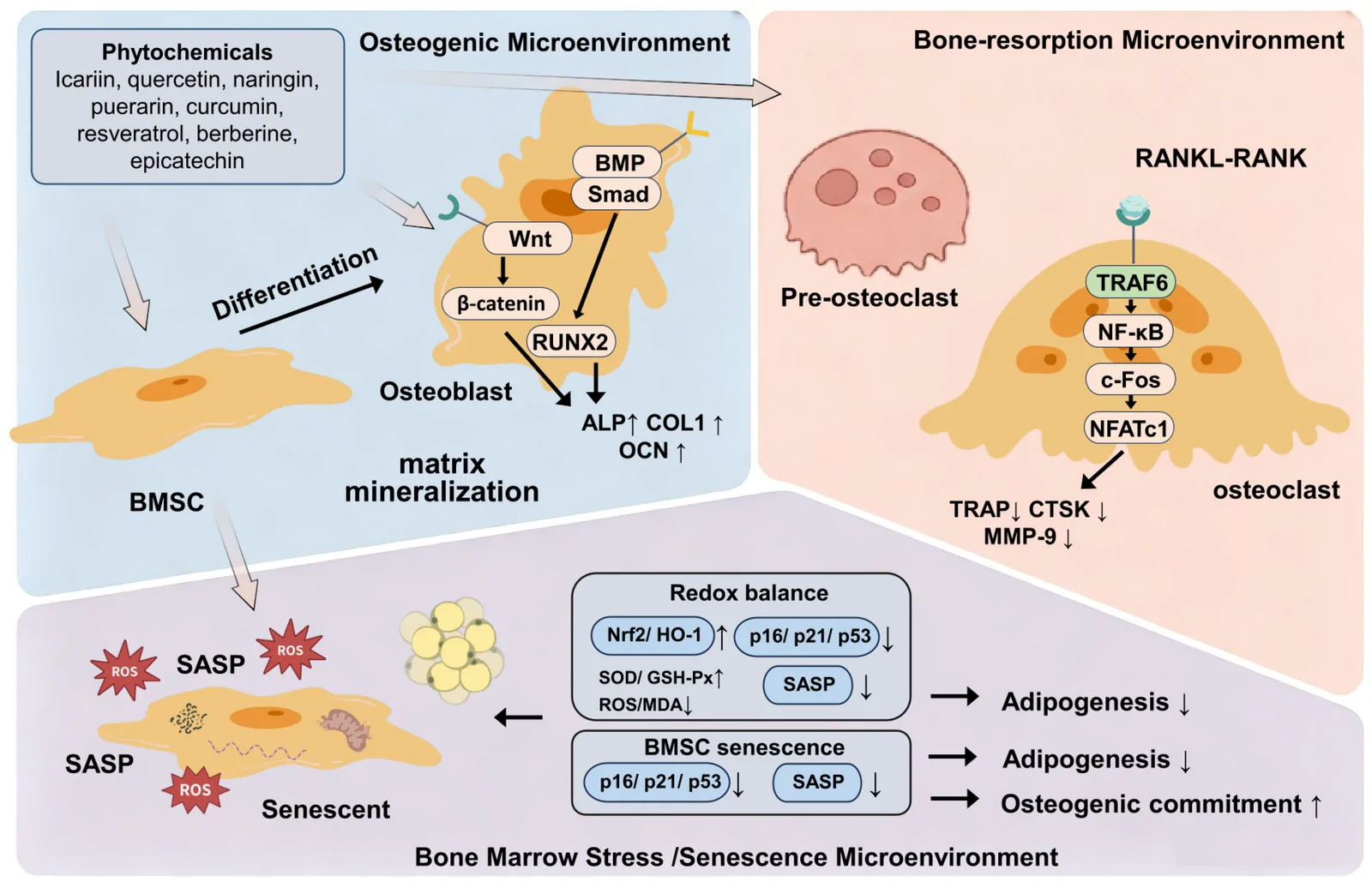
Phytochemicals regulate osteogenesis, osteoclastogenesis, oxidative stress, senescence, and marrow adipogenesis in estrogen-deficiency osteoporosis. Created with BioGDP.com [32].

Representative cargoes can be grouped according to their principal site of action. The first category is primarily osteogenic, promoting the osteogenic differentiation of bone marrow mesenchymal stem cells (BMSCs) and matrix mineralization through bone morphogenetic protein (BMP)/Smad and Wnt/β-catenin–runt-related transcription factor 2 (RUNX2) signaling; typical examples include icariin/icaritin, naringin, and astragaloside IV. The second category is mainly anti-osteoclastogenic and anti-inflammatory, inhibiting osteoclast differentiation and bone resorption through receptor activator of nuclear factor-κB ligand (RANKL)–NF-κB signaling; representative compounds include curcumin and celastrol. The third category primarily targets oxidative stress and the senescent bone marrow microenvironment, improving BMSC function, limiting bone marrow adipogenesis, and contributing to senescent-cell clearance through nuclear factor erythroid 2-related factor 2 (Nrf2)/heme oxygenase-1 (HO-1) and p16/p21/p53–senescence-associated secretory phenotype (SASP) signaling; representative compounds include quercetin, resveratrol, and epicatechin. In addition, puerarin, daidzein, and genistein exhibit phytoestrogen-like activity, linking their bone-protective effects to endocrine signaling (Figure 3).

### 2.1. Flavonoids and Isoflavones

Flavonoids and isoflavones are the most extensively studied phytochemicals in PMOP/EDO-related nanomedicines. Among them, icariin, quercetin, naringin, and puerarin are consistently supported in the literature for bone protection in OVX/PMOP models, including improvements in bone mass, bone mineral density (BMD), trabecular architecture, or bone turnover markers; they therefore constitute the core cargoes of this review (Table 1) [29,33,34,35,36,37,38]. Although the bone-protective activity of these compounds is relatively well established, their effects are difficult to reproduce consistently when they are administered as free drugs, owing to limited aqueous solubility, insufficient metabolic stability, and poor skeletal targeting [21,36]. This is also the main reason these compounds have been incorporated into nanodelivery and surface-engineering designs.

Among isoflavones, besides puerarin, genistein and daidzein have also been used in studies of estrogen deficiency or bone regeneration [44,45]. Kaempferol has shown some potential in PMOP-related studies addressing BMSC senescence, mitochondrial function, and osteogenic regulation [46]. At present, baicalein/baicalin and formononetin are supported mainly by indirect or non-PMOP evidence and are therefore mentioned only briefly, as supplementary examples [43].

### 2.2. Curcuminoids and Stilbenes

Curcumin and resveratrol are representative cargoes of curcuminoids and stilbenes, respectively. Curcumin exhibits multiple activities in osteoporosis models, including osteogenic, anti-osteoclastogenic, anti-inflammatory, and antioxidant effects [40], but its use is limited by poor aqueous solubility, poor chemical and photostability, and extensive metabolism; human pharmacokinetic data indicate that plasma levels of free curcumin remain very low even with absorption-enhancing formulations [41]. This review therefore treats curcumin as an example of a delivery bottleneck: its delivery limitations are well documented in both preclinical and human data, and it is frequently used to test engineered delivery strategies. Resveratrol can improve bone strength, trabecular parameters, and bone quality-related indicators in OVX rats [42], but it has low oral bioavailability, rapid metabolism, and variable tissue exposure.

### 2.3. Shared Delivery Barriers and Review S Cope

Overall, these compound classes face consistent delivery barriers: poor aqueous solubility, low oral bioavailability, limited metabolic stability, extensive first-pass metabolism, and rapid in vivo clearance. Together, these factors make skeletal exposure difficult to predict. Beyond these core categories, other phytochemicals appear as cargoes in studies of specific nanoplatforms: triterpenoids and saponins, such as astragaloside IV, celastrol, and pomolic acid; alkaloids, such as berberine; sesquiterpenes, such as dihydroartemisinin; iridoid glycosides, such as geniposidic acid; chlorogenic acid; and epicatechin. Because they share similar delivery barriers, these compounds are not discussed individually here but are addressed in Section 3 in relation to their specific nanoplatforms and surface-engineering strategies. Given that simply increasing systemic exposure does not ensure effective local concentrations within spatially heterogeneous skeletal target regions [21,22,23], Section 3 examines the relevant nanocarrier platforms and their mineral-affinity, cell-affinity, and pathology-responsive strategies, whereas Section 4 addresses imaging-supported evaluation of in vivo distribution and therapeutic outcomes.

## 3. Nanodelivery Platforms and Surface/Interfacial Engineering Strategies for Phytochemicals in OVX/PMOP-Related Models

**Figure 4:**
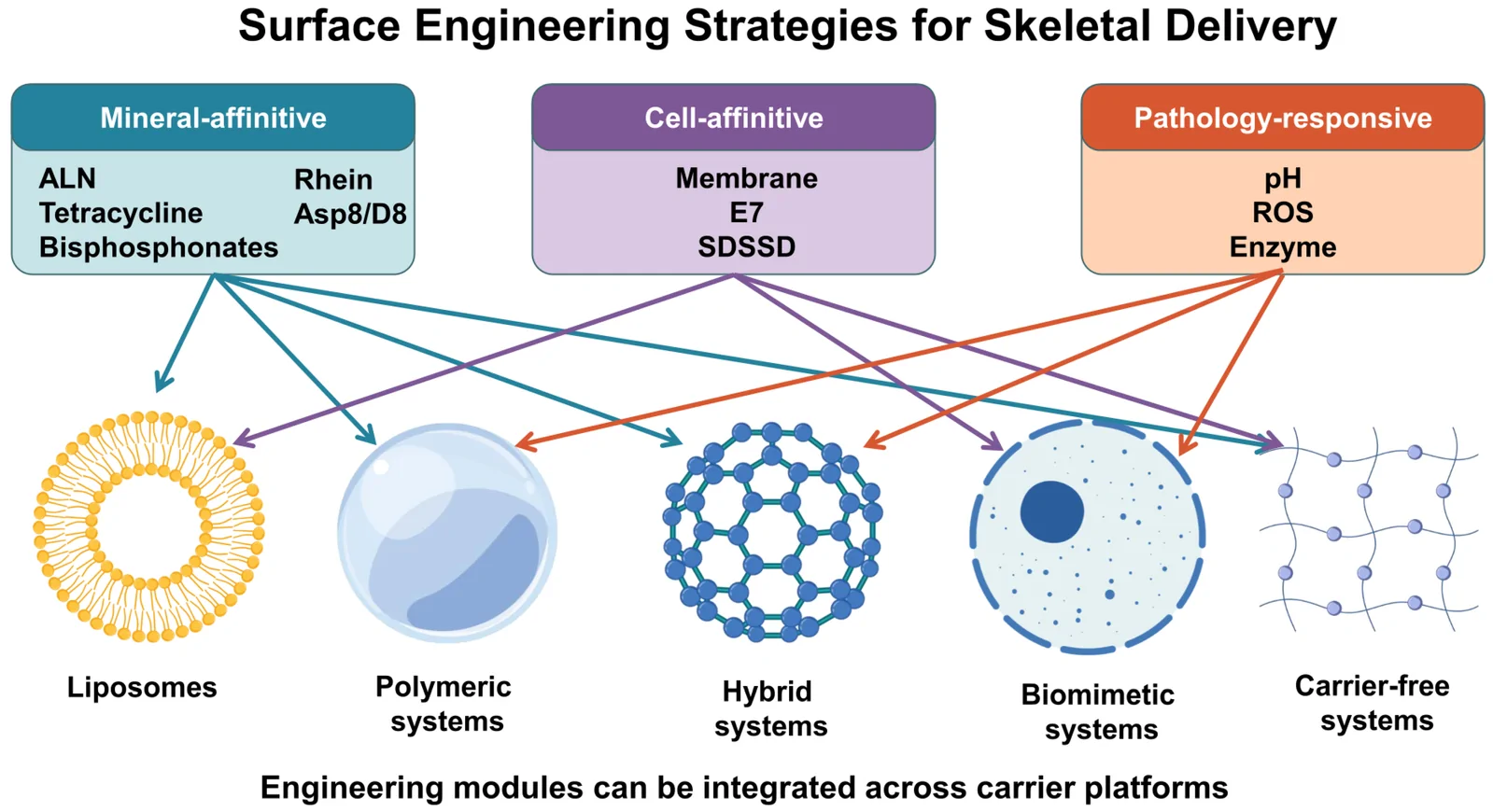
Mineral-affinity, cell-affinity, and pathology-responsive modules can be integrated across different nanocarrier platforms for skeletal delivery. Created with BioGDP.com [32].

Nanoplatforms for phytochemical delivery are diverse, but their skeletal delivery performance is mainly governed by two relatively independent dimensions: carrier structure and surface/interfacial engineering strategy. According to carrier structure, representative systems can be divided into five categories: lipid-based systems, polymeric/polyphosphazene systems, organic–inorganic hybrid systems, biomimetic membrane/cell-affinity interface systems, and carrier-free/supramolecular systems. By engineering strategy, they fall into mineral-affinity, cell-affinity, and pathology-responsive categories. These two dimensions do not correspond one to one: for example, alendronate (ALN)-mediated mineral affinity can be applied to multiple platforms, whereas a single platform such as liposomes can also carry multiple strategies (Table 2; Figure 4). In interpreting comparative results across studies, this review distinguishes overall formulation-level benefits from the incremental contribution of surface/interfacial engineering. When a nanoformulation is compared only with the free compound, the observed improvements in solubility, stability, bioavailability, circulation time, systemic exposure, or therapeutic outcome are first interpreted as benefits arising from nanonization or the overall formulation design. The most direct support for an additional contribution of surface/interfacial engineering itself typically comes from a non-targeted carrier control that is matched as closely as possible in composition and key formulation parameters and lacks only the intended surface module, combined with quantitative evidence capable of revealing differences in delivery. Different readouts support different conclusions: HAp binding primarily indicates in vitro mineral affinity; fluorescence/NIR can indicate carrier- or probe-associated bone-related distribution; and bone-tissue drug concentration, quantitative tissue distribution, or, under an appropriate design, pharmacokinetics combined with radiotracing can more directly support differences in actual exposure. Accordingly, for studies that lack a matched non-targeted control or provide only in vitro mineral affinity, carrier/probe imaging, or comparison with the free drug, this review still retains their value as evidence of formulation improvement, bone-related distribution, or preliminary targeting, but does not attribute all observed gains directly to surface engineering itself.

### 3.1. Lipid-Based Platforms

Lipid-based nanocarriers are among the earliest used and more pharmaceutically mature platforms in osteoporosis models and can be divided by function. Solid lipid nanoparticles (SLNs), nanostructured lipid carriers (NLCs), and nanosuspensions are mainly used for solubilization and stabilization: quercetin SLNs and naringenin nanosuspension/lipid systems optimize the particle size, ζ potential, encapsulation efficiency, and release behavior of poorly soluble flavonoids, yielding formulations suitable for in vivo evaluation [57,58,61,62]. Because these systems do not carry bone-directed interfaces, they mainly reflect the benefits of nanonization.

Liposomes have modifiable membrane structures, making them the most commonly surface-engineered lipid platform, and they can present three types of mineral-affinity modules. Tetracycline derivatives can bind Ca^2+^-rich mineral surfaces [31]; bisphosphonates such as ALN can chelate Ca^2+^ on hydroxyapatite (HAp) surfaces through their P–C–P backbone [63]; and aspartic acid-rich peptides can bind HAp through carboxyl groups, with binding capacity adjustable through sequence design [64]. In phytochemical delivery systems, icariin-loaded tetracycline/1,2-distearoyl-sn-glycero-3-phosphoethanolamine-poly(ethylene glycol) (DSPE-PEG) liposomes showed better bone-associated distribution and in vivo exposure than free drug or non-targeted liposomes, a result jointly supported by HAp-binding assays, fluorescence imaging, and pharmacokinetics [26]. ALN-functionalized liposomes loaded with the triterpenoid pomolic acid increased bone accumulation and attenuated bone loss in OVX mice [65], whereas Asp8-modified icaritin-loaded liposomes promoted bone formation in OVX mice [47]. The same acidic peptide strategy is not limited to liposomes: Asp8-modified carboxymethyl chitosan nanoparticles also showed strong HAp affinity and improved bone structure in OVX mice [66], supporting the cross-platform transferability of surface-engineering modules.

In addition to mineral affinity, liposomes can present cell-affinity ligands such as the BMSC-affinity peptide E7, or be designed as long-circulating or pathology-responsive systems. D-α-tocopheryl polyethylene glycol 1000 succinate (TPGS)-modified puerarin long-circulating liposomes mainly enhanced systemic exposure and bioavailability, an effect closer to nanonization [22]; in contrast, (DSS)_6_-modified quercetin liposomes extended lipid delivery toward bone-targeted regulation of the senescence-associated bone marrow microenvironment [67]. Among lipid platforms, therefore, SLNs and nanosuspensions are best understood as solubilizing formulations, whereas the distinctive feature of liposomes is their ability to carry multiple interfacial modules, including tetracycline, bisphosphonates, acidic peptides, and cell-affinity peptides. When matched controls are incorporated into the study design, liposomes are more useful for revealing the incremental contribution of surface engineering.

### 3.2. Polymeric and Polyphosphazene Platforms

Compared with most lipid systems, polymeric nanocarriers have higher structural tunability. Poly(lactic-co-glycolic acid) (PLGA), poly(ethylene glycol)-poly(ε-caprolactone) (PEG-PCL), polymeric micelles, polyphosphazene backbones, and prodrug polymers can be designed by adjusting composition, molecular weight, hydrophilic–hydrophobic balance, degradation rate, and functional groups, making them well suited for controlled release, ligand conjugation, and responsive release.

At the formulation level, these platforms can provide a basis for stable loading and controlled release of poorly soluble cargoes. For example, PLGA nanoparticles, degradable polymeric nanoparticles, and PEG-PCL micelles have been used to deliver curcumin, a quercetin/curcumin combination, and nobiletin, respectively [60,68,69], thereby improving in vivo accessibility and showing protective effects or sustained-release potential in OVX-induced bone loss. As with lipid SLNs, these effects mainly reflect nanonization.

Conjugating bone-affinity groups can enhance distribution, with ALN modification being the most common design. ALN-functionalized micelles loaded with icaritin, myricetin, or astragaloside IV showed better HAp affinity and bone-tissue concentrations than non-targeted micelles, while also improving oral bioavailability and enhancing antiresorptive activity [49,50,51]. Tetracycline provides a complementary and more compact bone-affinity module: tetracycline-grafted methoxy poly(ethylene glycol)-poly(lactic-co-glycolic acid) (mPEG-PLGA) micelles delivering astragaloside IV also increased accumulation in bone tissue in OVX rats and improved bone mass-related outcomes [70].

Polyphosphazene and prodrug systems represent more integrated designs that can combine cargo loading, bone affinity, and responsive release within a single structure. Polyphosphazene backbones have been used to deliver quercetin, curcumin, and resveratrol [24,54,71], coupling ALN-mediated or self-assembled bone affinity with acid-responsive polyphosphazene chemistry; among these, the resveratrol self-framed system suggests that non-flavonoid stilbenes can also participate in constructing such structures. The prodrug route further covalently integrates active compounds into responsive backbones, such as hyperbranched polyglycerol–curcumin prodrugs [48]. The defining feature of this platform is therefore structural designability, namely the capacity to integrate controlled release, conjugation, degradation, and responsive chemistry within one framework. This makes it particularly suitable for cargoes that are poorly soluble, rapidly metabolized, or require local release control.

### 3.3. Organic–Inorganic Hybrid Platforms

In organic–inorganic hybrid platforms, inorganic components not only provide structural support but can also confer mineral affinity, pH responsiveness, ion release, or redox activity. The contribution of such platforms is therefore not limited to increasing drug-loading capacity; it also includes modulation of the bone microenvironment.

Metal–organic framework (MOF) and carbonate systems illustrate this feature. Cur@ZIF@Asp8 integrates curcumin loading, zeolitic imidazolate framework-8 (ZIF-8)-mediated pH-responsive degradation, and Asp8-mediated mineral affinity within a single framework [27]. An amorphous calcium carbonate (ACC)-based wogonin A system uses its ACC core to neutralize osteoclast-associated acidic niches and achieves bone-associated interfacial interaction and release through a hexa-glutamic acid (Glu6)-modified layer [72]. In both systems, the carrier simultaneously provides cargo protection, bone-associated localization, and pathology-responsive regulation.

Mesoporous inorganic carriers have high specific surface area and controlled-release capacity. Bone-targeted mesoporous silica delivering celastrol inhibited osteoclast formation and improved trabecular outcomes in OVX rats [55]. Mesoporous silica delivering dihydroartemisinin maintained BMSC stemness and improved bone-associated therapeutic effects [73]. Ipriflavone-loaded SiO_2_–CaO bioactive nanoparticles were mainly used for the repair of osteoporotic bone defects [74,75], a regenerative scenario related to but distinct from systemic OVX/PMOP delivery. HAp-based systems have intrinsic bone affinity because of their similarity to bone mineral: daidzein-loaded HAp nanoparticles provided a basis for menopause-related HAp–isoflavone delivery, supplemented by genotoxicity and pharmacokinetic data [56]. In addition, a salvianic acid A system with an HAp core and an ALN-PEG-polysuccinimide shell showed that mineral affinity can be jointly constructed by an inorganic core, an outer polymer layer, and additional bone-affinity ligands [63].

Inorganic interfaces can also exploit natural small molecules or ion release. Because of its bone-mineral affinity, rhein can serve as a linker to construct rhein-PEG-nano-HAp conjugates [76]. Quercetin-capped selenium nanoparticles combine flavonoid activity with an inorganic interface [77]. Epicatechin-containing silicate/Mn nanomedicines can release Mn^2+^, epicatechin, and silicate in acidic osteoporotic regions, coupling antioxidant activity with ion-mediated inhibition of bone resorption and promotion of mineralization [78]. The distinctive contribution of hybrid platforms therefore lies in the ability of the carrier itself to buffer acidity, release ions, or interact with minerals, thereby matching multiple compartments involved in osteoporosis, including the mineralized matrix, bone resorption microdomains, and bone marrow cells.

### 3.4. Biomimetic Membrane and Cell-Affinity Interface Systems

Biomimetic membranes and cell-affinity interfaces shift the targeting focus from minerals to cellular recognition. Bone loss involves not only the mineralized matrix but also osteoblasts, osteoclasts, BMSCs, endothelial cells, and immune-related bone marrow components. Interfaces capable of presenting membrane proteins, adhesion molecules, chemokine receptors, or recognition peptides can therefore enhance the association between carriers and bone marrow or bone-remodeling niches [79,80].

Cell membrane coating is the most information-rich interface. Osteoblast-like cell membrane-coated curcumin-loaded PLGA nanoparticles (OM/Cur@NPs) combine PLGA-mediated curcumin delivery with membrane C-X-C chemokine receptor type 4 (CXCR4) and tumor necrosis factor-α (TNF-α) receptors. Compared with uncoated nanoparticles, they showed higher bone accumulation and simultaneously modulated the inflammatory microenvironment [40], providing a relatively clear non-targeted carrier control. Although a separate synthetic system combining a cell-membrane-mimicking, bone-targeting interface with ROS-controlled release did not carry a phytochemical cargo, it suggests that biomimetic interfaces can be combined with ROS-responsive design [81], a concept that may be further extended to phytochemicals with antioxidant or osteogenic activity.

Cell-affinity interfaces can also use peptide ligands that have better-defined compositions and are easier to standardize. E7-modified apigenin liposomes use BMSC recognition to increase apigenin enrichment in bone and improve bone mass-related indicators in OVX mice, extending the delivery focus from bone mineral surfaces to marrow niches [53]. The osteoblast-targeting peptide SDSSD–geniposidic acid system (SGPA) accumulates in osteoblasts, promotes bone formation, and activates farnesoid X receptor (FXR)–RUNX2 signaling in OVX mice [52]. Compared with mineral-affinity peptides such as Asp8 and D8, these ligands place greater emphasis on spatial association with bone marrow cells or osteoblast regions. Membrane coating and peptide ligands are therefore complementary: membrane interfaces are information-rich but structurally complex, whereas peptide ligands have defined compositions but narrower recognition ranges. Both indicate that phytochemical delivery can move beyond mineral surfaces toward bone marrow cellular niches, although cell-level localization within the marrow still requires confirmation by section-level or single-cell evidence.

### 3.5. Carrier-Free and Supramolecular Systems

In carrier-free and supramolecular systems, the active molecules themselves participate in nanostructure formation and can provide mineral affinity or responsiveness. Reducing inert excipients, increasing drug loading, and simplifying the structural relationship between cargo and carrier give these designs practical significance for chronic diseases such as PMOP that require long-term administration.

Molecular phosphorylation is a form of endogenous interfacial engineering. Phosphorylation of the phenolic hydroxyl groups of quercetin, curcumin, and dihydromyricetin can simultaneously improve dispersibility, confer bone-mineral affinity, and increase drug-loading capacity [25,82,83]. Among these, phosphorylated dihydromyricetin can self-assemble into nanostructures in one step, inhibit osteoclast differentiation in vitro through the NF-κB/mitogen-activated protein kinase (MAPK) pathway, and attenuate bone loss in OVX mice more effectively than the free drug. Phosphorylation can therefore not only solubilize polyphenols but also convert them into bone-affinity structural building blocks.

Supramolecular self-assembly provides another route. A berberine/chlorogenic acid supramolecular nanoagonist self-assembles through non-covalent interactions between herb-derived components, achieves high drug loading, and promotes bone formation in OVX mice through Wnt-related mechanisms [59]. An icariin-loaded para-phosphonated calixarene constructed an HAp-responsive system in which the phosphonate interface was not an added ligand but part of the supramolecular scaffold itself [84]. In addition, cucurbituril–chlorogenic acid nanocomplexes improved the biocompatibility and release behavior of chlorogenic acid while preserving its antioxidant activity [85]. These systems show that macrocyclic host–guest interfaces can provide both structure and bone affinity.

Carrier-free and supramolecular systems are therefore characterized by low inert excipient content and the participation of cargoes in structure formation, making them suitable for high-drug-loading and bone-affinity designs. However, as with other platforms, their bone affinity still needs to be verified against matched in vivo controls.

### 3.6. Cross-Platform Comparison of Surface/Interfacial Engineering Strategies and Platform Selection

From a cross-platform perspective, bone-targeted surface engineering is a design space rather than a single technology. This design space includes mineral-affinity, cell-affinity, and pathology-responsive strategies, which differ in platform compatibility, maturity, and applicable scenarios.

Mineral-affinity strategies are currently the most widely applied. ALN and other bisphosphonate/phosphonate groups are chemically well defined and have strong HAp-binding capacity; they have been applied to liposomes, polymeric micelles, polyphosphazene backbones, HAp-based hybrid systems, and supramolecular systems. Acidic peptides such as Asp8/D8 have sequence tunability and can be optimized for binding capacity and surface exposure, whereas compact modules such as tetracycline and rhein further expand the repertoire of bone-affinity ligands.

Cell-affinity strategies shift the targeting focus from minerals to bone marrow and cellular niches. Membrane coating can provide a multimolecular recognition interface and support tissue homing, cellular recognition, and microenvironmental modulation. In contrast, well-defined peptides such as E7 and SDSSD can be used to construct BMSC- or osteoblast-directed systems. These strategies are relatively well matched with phytochemicals that act on BMSC differentiation, oxidative stress, or the bone marrow microenvironment.

Pathology responsiveness is a cross-platform dimension of release control. Osteoclast-associated acidic microdomains, elevated oxidative stress, and matrix-degrading enzymes, including cathepsin K (CTSK) and matrix metalloproteinase-9 (MMP-9), in osteoporotic bone tissue provide a biological basis for pH-, ROS-, and enzyme-responsive release [86,87]. Among these, pH responsiveness is the most mature and has been applied in polyphosphazene, ZIF-8, calcium carbonate, and prodrug systems. ROS- and enzyme-responsive designs remain more exploratory and mainly focus on bone marrow oxidative stress and osteoclast niches [88].

Platform selection can also be matched to specific requirements. Liposomes suit ligand decoration and diverse surface modifications; polymeric and polyphosphazene systems suit the integration of controlled release, conjugation, and responsive chemistry; hybrid systems suit mineral similarity, acid buffering, ion release, and redox regulation; biomimetic and peptide interfaces suit linkage to marrow niches; and carrier-free/supramolecular systems suit reducing the excipient fraction. Only when formulation parameters, biodistribution, tissue localization, and therapeutic outcomes are reported more consistently can surface engineering demonstrate its ability to improve bone-associated delivery and therapeutic performance in OVX/PMOP-related models.

## 4. Delivery Evaluation and Therapeutic Performance of Surface-Engineered Nanomedicines in OVX/PMOP-Related Models

**Figure 5:**
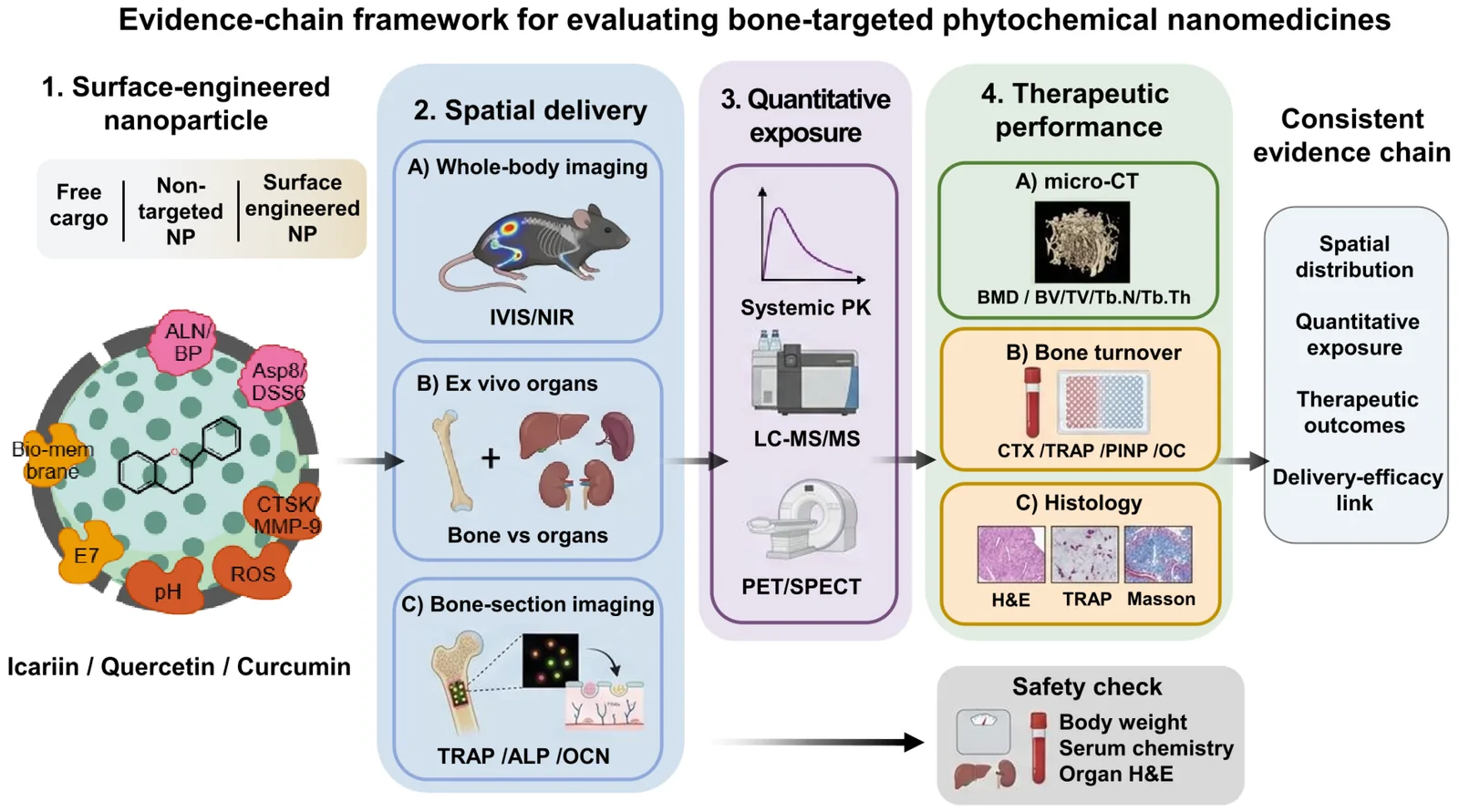
A multimodal evidence chain links surface engineering, spatial delivery, quantitative exposure, therapeutic outcomes, and safety evaluation. Created with BioGDP.com [32].

Evaluation of surface-engineered phytochemical nanomedicines usually involves three interrelated levels: first, the overall distribution of the carrier after administration and its bone-associated enrichment; second, at the tissue scale, the spatial relationship between the carrier and bone-remodeling regions, osteogenic/osteoclastic markers, or the bone marrow microenvironment; and third, whether these distribution and localization features are accompanied by improvements in bone structure, bone turnover, and histology. Imaging mainly addresses the first two levels, whereas micro-CT, bone turnover markers, and histology characterize therapeutic performance; safety observations provide information on preclinical tolerability (Table 3; Figure 5). Within this evaluation framework, different readouts address different questions: in vitro mineral affinity, carrier- or probe-associated distribution, tissue- or cell-level localization, quantitative cargo exposure, and post-treatment bone-structural outcomes should be interpreted separately rather than being merged into a single body of “bone-targeting evidence.”

### 4.1. Imaging-Supported Evaluation of Bone-Associated Distribution and Localization

In OVX/PMOP studies, imaging is used mainly to assess the in vivo distribution of nanosystems after administration and their spatial relationship with bone tissue or the local microenvironment. Fluorescence imaging with exogenous probes such as DiR and fluorescein isothiocyanate (FITC) or with labeled carriers, near-infrared (NIR) imaging, and IVIS are the most common methods. By comparing the signals of free drugs, non-targeted carriers, and surface-engineered carriers in bone regions, one can judge whether mineral-affinity ligands, cell-affinity peptides, or biomimetic interfaces alter the bone-associated distribution of the labeled system [22,26,89]. Ex vivo organ imaging can further compare the relative signal distribution between bone and major organs such as the liver, spleen, lung, and kidney, whereas time-series imaging, matched non-targeted carrier controls, and major-organ analysis improve the interpretation of the incremental contribution of surface modification.

At the probe level, optimized NIR probes can improve the quality of bone imaging: P800SO_3_-PEG shows high affinity for hydroxyapatite/calcium phosphate with low nonspecific uptake [90], whereas NIR-II and long-afterglow imaging reduce background and improve visualization of deep bone tissue [91,98]. However, such advances enhance the sensitivity and spatial quality of signal detection rather than conferring analyte specificity; the signal still reflects the labeled probe or carrier rather than the phytochemical cargo itself.

It should be emphasized that when fluorescence or NIR signals originate from carrier labeling or exogenous probes, they primarily reflect carrier- or probe-associated distribution and bone-region enrichment, and cannot by themselves demonstrate an increase in the concentration of the actual phytochemical cargo in bone tissue. Even when fluorescence intensity is quantified by region-of-interest analysis, the result remains a quantification of signal rather than an analyte-specific measurement of cargo concentration. More direct assessment of actual cargo exposure can be obtained using analyte-specific bone-tissue concentration measurements such as LC-MS/MS, or tissue pharmacokinetics. Plasma pharmacokinetics mainly reflects systemic exposure and cannot by itself demonstrate a local increase in cargo concentration in bone.

Radiotracing, PET, and SPECT can provide quantitative in vivo distribution information for the labeled species, but their interpretation depends on whether the label is attached to the cargo, the carrier, the surface ligand, or a surrogate tracer, and on whether it remains stably associated with that component in vivo. These methods therefore strengthen quantitative biodistribution assessment but cannot be equated with actual cargo exposure without appropriate validation of the labeling strategy.

Compared with whole-body or ex vivo organ imaging, bone-section confocal imaging can further resolve the spatial relationship between carrier or probe signals and the local bone microenvironment. Tartrate-resistant acid phosphatase (TRAP), cathepsin K (CTSK), or matrix metalloproteinase-9 (MMP-9) can be used to evaluate the spatial association between signals and bone-resorption regions or osteoclast-related microenvironments [86,92], whereas alkaline phosphatase (ALP), osteocalcin (OCN), RUNX2, and COL1, together with BMSC or senescence-related markers, help describe their relationship with osteogenic regions or the bone marrow niche [67]. However, spatial proximity or co-localization on sections mainly supports tissue-level or cell-associated localization; unless cellular internalization or cell-specific uptake is further validated, it should not be interpreted as intracellular cargo exposure or target binding.

Accordingly, different evaluation methods address complementary but non-interchangeable questions: HAp binding primarily reflects in vitro mineral affinity; fluorescence and NIR imaging primarily describe carrier- or probe-associated distribution; bone-section imaging provides tissue-level or cell-associated localization; analyte-specific tissue-concentration measurement more directly assesses actual cargo exposure; and appropriately designed radiotracing, PET, or SPECT provides quantitative distribution information for the labeled species. Correspondingly, micro-CT mainly evaluates post-treatment bone-structural outcomes rather than delivery itself.

### 4.2. Therapeutic Performance: Bone Structure, Bone Turnover, and Histology

Micro-CT is the core method for assessing improvements in bone structure in OVX/PMOP studies. BMD, bone volume/tissue volume (BV/TV), trabecular number (Tb.N), trabecular thickness (Tb.Th), trabecular separation (Tb.Sp), and cortical bone parameters reflect bone mass and bone microarchitecture and are basic indicators for comparing different formulations [93,94]. Unlike fluorescence or NIR imaging, micro-CT reports structural outcomes after treatment; it can support the functional relevance of a surface-engineering strategy only when the direction of improvement is consistent with bone-associated distribution, tissue localization, or quantitative exposure. Phytochemical nanoformulations can improve bone mass and trabecular architecture to varying degrees. Compared with free naringenin and raloxifene, naringenin nanosuspensions improved BMD, bone mineral content, and bone histology in OVX rats [58], and quercetin/curcumin co-loaded polymeric nanoparticles also enhanced anti-osteoporotic effects [68]. These benefits mainly reflect the overall exposure advantage conferred by nanonization. The added value of surface engineering can be better demonstrated when mineral-affinity, cell-affinity, or pathology-responsive systems show both stronger bone-associated distribution and more marked structural improvement than matched non-targeted carriers.

Bone-affinity or responsive designs provide more direct evidence linking improved delivery with therapeutic efficacy. Phosphorylated quercetin improved dispersibility, bone affinity, and bioavailability while attenuating trabecular bone loss in OVX mice [25]. Bone-targeted celastrol nanocarriers improved BMD, preserved trabecular architecture, and reduced osteoclast numbers in OVX rats [55]. Cur@ZIF@Asp8 combines curcumin loading, ZIF-8 pH responsiveness, and Asp8-mediated mineral affinity, and showed outcomes consistent with bone delivery and improved bone homeostasis [27]. In these examples, surface engineering mainly makes bone-associated exposure and local action more observable and easier to link to outcomes; however, in most cases, the quantitative therapeutic gain over matched non-targeted carriers has not been fully isolated [99].

Bone turnover markers can indicate whether therapeutic efficacy favors inhibition of bone resorption, promotion of bone formation, or restoration of bone-remodeling balance: type I collagen C-terminal telopeptide (CTX-I), TRAP, and TRAP-5b reflect bone resorption; procollagen type I N-terminal propeptide (PINP/P1NP), osteocalcin (OCN), and bone-specific alkaline phosphatase (BALP) reflect bone formation; and RANKL/osteoprotegerin (OPG) reflects bone-remodeling balance [29,34,94]. These indicators complement the structural data from micro-CT by adding dynamic metabolic information. When structural improvement is accompanied by reduced TRAP/CTX-I or increased PINP/OCN/BALP, the mode of action of a formulation can be described more clearly.

Histology helps explain these structural and metabolic changes: hematoxylin and eosin (H&E) staining reveals trabecular continuity and bone marrow morphology; TRAP staining shows osteoclast burden; Masson/Goldner staining shows bone matrix formation and mineralization; and calcein double labeling captures dynamic bone formation. For systems acting on BMSC function, bone marrow adipogenesis, or senescence, adding endpoints such as bone marrow fat content, BMSC osteogenic/adipogenic balance, or senescence markers helps clarify how impaired osteogenesis and marrow changes influence the PMOP treatment response [95].

Therapeutic performance is therefore mainly characterized by micro-CT, bone turnover markers, and histology, whereas imaging and localization explain how distribution is related to local action. The strongest conclusions come from the convergence of structural, metabolic, and localization evidence, and this convergence supports the functional link between a surface-engineering strategy and its therapeutic effect.

### 4.3. Safety Observations in Preclinical Studies

Safety evaluation in OVX/PMOP nanomedicine studies currently centers on preliminary tolerability indicators: body weight, hematology, liver and kidney function, serum biochemistry, and H&E staining of major organs (heart, liver, spleen, lung, and kidney), used to detect overt systemic toxicity or organ pathology after short-term administration. It should be emphasized that these indicators reflect only preliminary tolerability under specific dosing conditions and are insufficient to support conclusions regarding long-term safety; nor can bone-associated enrichment itself be automatically interpreted as reduced extraosseous exposure or improved safety. Some studies also include ex vivo organ imaging or biodistribution analysis to provide preliminary observations of extraosseous carrier signals [100,101,102].

For surface-engineered systems, safety data are best interpreted in relation to carrier material and modification type. Acidic peptides, bisphosphonate/phosphonate groups, biomimetic membranes, and inorganic components can all affect hemocompatibility and organ distribution. Hybrid systems such as ZIF-8, amorphous calcium carbonate, and silicate/Mn can provide pH responsiveness or ion release, but their biocompatibility depends on composition and therefore usually needs to be reported together with material details [103,104]. For phytoestrogen-like cargoes such as genistein and daidzein, some studies have begun to examine endocrine endpoints such as uterine responses [105,106]. Current OVX safety data therefore mainly describe short-term tolerability. Long-term repeated administration, quantitative extraosseous accumulation, and cargo-specific safety remain issues that need to be addressed at the translational stage, and together with these short-term observations they form a continuous safety evidence chain.

## 5. Translational Considerations and Future Perspectives

Surface-engineered phytochemical nanomedicines have already improved bone-associated distribution, local exposure, and therapeutic performance in OVX/PMOP-related models. Advancing these benefits toward translation depends on formulation reproducibility, model relevance, long-term safety, and clinical positioning. Unlike conventional small molecules, the in vivo behavior of these systems is determined not only by the cargo but also by carrier material, particle size, surface charge, ligand density, serum stability, and release behavior. Future progress will therefore depend on whether the reproducibility, interpretability, and translational relevance of delivery effects can be set as explicit design and reporting goals (Table 4).

### 5.1. Formulation Reproducibility and Critical Quality Attributes

Formulation reproducibility is the basis for translating surface-engineered phytochemical nanomedicines. Particle size, polydispersity index (PDI), ζ potential, encapsulation efficiency, drug loading, release profile, serum stability, storage stability, and batch-to-batch consistency all affect circulation behavior, tissue distribution, bone-associated enrichment, and therapeutic effects [107,108]. For phytochemicals with poor solubility and low bioavailability, nanonization can improve dispersibility, stability, and systemic exposure. However, if particle size, encapsulation efficiency, or release behavior varies substantially between batches, differences in therapeutic efficacy across animal studies will be difficult to interpret [109,110]. Surface engineering further complicates quality control. Introducing bone-targeting ligands, PEG modification, biomimetic membranes, or inorganic interfaces can alter particle size, charge, surface hydrophilicity, serum protein adsorption, and clearance routes. For example, introducing ALN through pre-conjugation or post-conjugation can produce different hydroxyapatite-binding capacities and bone-enrichment characteristics [111,112].

Ligand density is a particularly important variable. Higher ligand density can enhance HAp binding, but it can also alter ζ potential, reduce colloidal stability, increase serum protein adsorption, or promote hepatic and splenic clearance. Bone affinity is therefore not simply determined by whether a ligand is introduced; instead, it reflects the combined effects of ligand density, spatial exposure, PEG shielding, surface conformation, and release behavior [89,113].

HAp-binding assays remain practical methods for evaluating mineral affinity, but they should be interpreted in relation to specific experimental conditions. Because different studies use HAp powder, HAp discs, or bone sections, and because incubation time, serum conditions, and washing stringency differ, binding percentages are usually difficult to compare directly across systems [114]. HAp binding reflects the mineral affinity of a material, whereas in vivo bone distribution, marrow-cavity exposure, and cellular uptake are also governed by circulation, clearance, and microenvironmental factors. Bone-affinity experiments are therefore best interpreted together with release behavior, serum stability, biodistribution, and tissue localization [115,116,117].

### 5.2. Model Relevance and Therapeutic Positioning

OVX rats and mice remain the main preclinical models of estrogen-deficiency-associated bone loss and are suitable for early screening of bone-protective effects, bone distribution characteristics, and differences among formulations. They usually show reduced BMD, decreased BV/TV and Tb.N/Tb.Th, and increased Tb.Sp at sites such as the proximal tibia, femur, and lumbar spine [93,118], making them suitable for comparing the protective effects of different delivery systems on bone structure.

However, human PMOP is a chronic, slowly progressive disease with marked interindividual variation and is affected by age, metabolic status, medication history, and comorbidities. Bone loss in OVX models occurs over weeks to months, whereas the clinical course usually extends over years; the timing of treatment initiation therefore affects interpretation of results. Administration soon after OVX resembles preventive intervention and is used to test whether a system can slow rapid bone loss after estrogen deficiency. Delayed-treatment OVX models are closer to intervention after low bone mass or microarchitectural damage has already formed and are more suitable for evaluating repair or partial reversal [110,119].

Complementary models can narrow the gap with human bone metabolism: aged OVX models more closely resemble the background of older PMOP, delayed-treatment OVX models simulate intervention after bone loss, and human BMSC or ex vivo bone models can address species differences. Across studies, animal age, OVX duration, treatment initiation time, skeletal site, and baseline bone mass remain key factors affecting comparability.

In terms of therapeutic positioning, surface-engineered phytochemical nanomedicines should be understood as platforms that improve the in vivo delivery and bone-associated exposure of natural compounds with low bioavailability, rather than as replacements for existing therapies such as bisphosphonates, denosumab, or anabolic agents. Their value is mainly reflected in improving the accessibility of poorly soluble or rapidly metabolized phytochemicals and in increasing bone-associated distribution through mineral-affinity, cell-affinity, or pathology-responsive structures; whether they can concurrently reduce off-target tissue exposure remains to be verified by quantitative distribution studies. This positioning is consistent with the current evidence base [120]. It should further be emphasized that the current evidence derives mainly from OVX rodent models, that the aged and delayed-treatment models more relevant to the clinical setting remain relatively limited, and that clinical validation is still lacking; key issues such as long-term repeated-dose tolerability, quantitative extraosseous accumulation, and clearance kinetics likewise remain to be resolved.

This positioning becomes clearer within the broader landscape of bone-targeted delivery. Parallel strategies include bisphosphonate–drug conjugates, bone-targeted nucleic acid systems, such as bone-affinity peptide-modified small interfering RNA (siRNA) nanoparticles, and biologics directed at defined targets, such as anti-RANKL and anti-sclerostin antibodies. These strategies usually have a single, defined target and a clear dose–response relationship, and some biologics already have clinical precedents [121]. However, most are single-pathway macromolecular systems and carry burdens related to cost, immunogenicity, or cold-chain requirements. The phytochemical route is different: its cargoes are multi-target, low-toxicity natural products that can act simultaneously on oxidative stress, inflammation, osteoclast activation, and insufficient osteogenesis; its interfaces can stack mineral-affinity, cell-affinity, and pathology-responsive modules to achieve delivery matched to bone-remodeling microregions; and most cargoes are relatively low cost and have potential for oral delivery. Its limitations are also clear: quantitative evidence for delivery gains remains relatively limited, system heterogeneity is high, and the field remains at the preclinical stage. This complementarity—that is, multi-target natural cargoes combined with stackable interfacial responsiveness, rather than single-target precision intervention—supports its role as an adjunctive platform rather than a replacement therapy [122,123].

### 5.3. Long-Term Safety, Extraosseous Accumulation, and Cargo-Specific Risks

Given the chronic nature of PMOP, safety evaluation cannot stop at short-term observations. Body weight, hematology, liver and kidney function, and H&E staining of major organs can provide preliminary tolerability information, but under long-term repeated administration, organ distribution, clearance routes, extraosseous accumulation, and tissue compatibility are also important, especially material residues in the liver, spleen, lung, kidney, and bone marrow reticuloendothelial system [97,124]. For bone-targeted nanomedicines, the ideal translational profile is to increase bone-associated exposure while limiting extraosseous accumulation. Quantitative distribution after long-term administration, major organ histology, and hematological and biochemical indicators are therefore core readouts.

Different surface-engineering modules raise different safety issues. Acidic peptides, bisphosphonate/phosphonate groups, tetracycline-related structures, and biomimetic membranes can alter protein adsorption, immune recognition, and clearance. Hybrid platforms such as ZIF-8, amorphous calcium carbonate, and silicate/Mn also involve scaffold degradation, metal ion release, and long-term residues [125,126,127]. The focus of long-term safety therefore varies with material composition. This does not weaken the value of these platforms but indicates that both safety evaluation and material design are platform-specific.

The cargo itself also affects safety. For phytoestrogen-like cargoes such as genistein, daidzein, and equol, safety is closely related to endocrine endpoints such as uterine weight and uterine histology [128,129]. For multi-target natural products such as curcumin, resveratrol, and quercetin, interactions among carrier materials, dosing frequency, and long-term exposure jointly shape the safety profile. Before judging whether these systems are suitable for long-term management of chronic disease, material-related safety, organ distribution, and cargo-specific safety need to be integrated [130].

### 5.4. Future Directions

Future designs can be organized around specific pathological processes in the bone microenvironment. PMOP simultaneously involves increased osteoclast activity, reduced BMSC osteogenic differentiation, greater cellular senescence, and bone marrow adipogenesis. Delivery targets deserving attention therefore include mineralized surfaces, bone resorption microdomains, osteoblast regions, and marrow niches. Interface strategies can then be matched to the cargo’s main action: anti-osteoclastogenic cargoes with bone resorption microdomains or acid-responsive designs; osteogenic or antioxidant cargoes with BMSC/osteoblast localization; and senescent-cell-clearing cargoes with defined endpoints centered on senescent marrow niches.

Delivery evaluation also needs to move progressively from a sole reliance on qualitative or probe-based signals toward more explicit quantitative exposure assessment. IVIS, NIR, ex vivo organ imaging, and bone-section confocal imaging can provide important temporal and spatial distribution information, but signal intensity by itself does not equate to the actual cargo concentration. When conditions allow, analyte-specific methods such as LC-MS/MS can be used to measure actual cargo exposure in bone tissue, whereas radiotracing, PET, or SPECT can provide quantitative biodistribution information for the labeled species, although their interpretation depends on the labeled component and the stability of the label. Future studies therefore need not adopt any single imaging or tracing technique mechanically, but should combine spatial imaging, analyte-specific quantification, and appropriate traceable-distribution methods in a complementary manner according to the research question, so as to distinguish more clearly between carrier distribution, cargo exposure, and therapeutic outcomes.

Co-delivery is a longer-term direction. Phytochemicals can be combined with nucleic acids, small molecules, peptides, antioxidants, or osteogenic/antiresorptive regulators to act simultaneously on oxidative stress, inflammation, osteoclast activation, and insufficient osteogenesis. Bone-targeted senescent-cell-clearing co-delivery of quercetin and dasatinib provides a methodological reference for senescence-associated bone loss [131,132,133]. However, such systems make interpretation more complex, and distinguishing single-agent effects, synergistic effects, and carrier effects remains a central challenge.

Maturation of this field will therefore depend less on adding more platforms and more on consistency in formulation parameters, model design, and delivery quantification across studies. When a surface-engineering strategy can show stable and interpretable delivery advantages across batches, models, and endpoints, these systems can move from conceptual bone-targeted platforms toward more reliable preclinical candidates.

## 6. Conclusions

Based on current evidence, surface-engineered phytochemical nanomedicines are best positioned as adjunctive platforms for improving the delivery, bone-associated exposure, and local action of natural compounds with low bioavailability, rather than as replacements for bisphosphonates, denosumab, or anabolic agents. Realizing this positioning will require more consistent reporting of formulation parameters—particularly ligand density, serum stability, release behavior, and batch-to-batch reproducibility—and validation of long-term efficacy and repeated-dose safety in aged or delayed-treatment OVX models that more closely approximate clinical PMOP.

Several limitations remain. Studies vary substantially in nanocarrier design, surface modification, imaging strategy, animal model, and outcome indicators, making cross-platform comparison difficult, and the evidence is still mainly preclinical and unevenly distributed across cargoes. The core question is therefore not whether bone-affinity nanoplatforms can be built, but whether surface engineering can generate delivery gains that are reproducible, interpretable, and translationally meaningful. HAp binding, fluorescence imaging, and micro-CT each address only part of this question—mineral affinity, carrier distribution, and structural outcome, respectively—so the most persuasive evidence comes from consistency across formulation parameters, biodistribution, tissue localization, cargo release, bone turnover markers, histology, and safety. When these lines of evidence form a coherent chain, the value of surface engineering goes beyond “adding a targeting ligand”: it helps explain why the same cargo, delivered through an engineered interface, can produce more reproducible bone-associated therapeutic effects.

## Figures and Tables

**Table 1:** Representative plant-derived phytochemical cargoes used in nanomedicines for postmenopausal/estrogen-deficiency osteoporosis. Compounds are listed from those with the most direct OVX/PMOP delivery evidence to those mentioned only briefly.

Cargo/Class [Ref]	Main Osteo-Action and Pathway	Evidence in OVX/PMOP Models	Key Delivery Limitation	Scope in This Review
Icariin/Herba Epimedii flavonoids [23,33]	Pro-osteogenic; BMP-2/Smad, Wnt/β-catenin–RUNX2	OVX rat; bone-protective and formulation/delivery studies	Low aqueous solubility; limited oral absorption; uncertain skeletal-site exposure	Core cargo
Quercetin [21,39]	Antioxidant/senolytic (Nrf2/HO-1, anti-SASP); anti-osteoclastic (NF-κB)	OVX osteoprotection; PK/formulation studies	Low oral bioavailability; extensive metabolism limits exposure	Core cargo
Naringin [34]	Pro-osteogenic; BMP/Wnt–RUNX2	OVX/PMOP rat; BMSC osteogenesis	Poor dissolution; oxidation-prone; unstable under acidic/enzymatic conditions	Core cargo
Puerarin [22,29,34]	ER-related signaling; pro-osteogenic, antioxidant	OVX/PMOP rat; long-circulating formulations	Short exposure window; insufficient skeletal-site concentration	Core cargo
Curcumin [40,41]	Anti-osteoclastic/anti-inflammatory (RANKL–TRAF6–NF-κB–NFATc1); antioxidant	Osteoporosis pharmacology; limited human PK	Poor solubility; chemical/photo-instability; very low free-plasma exposure	Core cargo (delivery-bottleneck exemplar)
Resveratrol [42]	Antioxidant (SIRT1); pro-osteogenic/anti-osteoclastic	OVX osteoprotection	Rapid metabolism; low, fluctuating tissue exposure	Core cargo
Baicalein/baicalin; formononetin [43]	Mixed osteogenic/anti-resorptive	Mostly indirect or non-PMOP evidence	Limited direct OVX/PMOP delivery evidence	–

**Table 2:** Representative phytochemical nanoformulations in OVX/PMOP-related models: platform/interface design, comparator, delivery evidence, and reported effect sizes (as reported in the original studies). Studies are grouped by comparator design (Groups A–C). Values are reported descriptively and are not pooled across studies.

Cargo/Platform · Interface Module [Ref]	Key Formulation Data	Comparator (s)	Delivery Evidence (Reported Value)	Therapeutic Effect (Reported Value vs. Comparator)	Evidence Level/Attribution
Group A—Matched non-targeted carrier available (incremental surface/interface effect can be isolated)					
Icaritin/Asp8 liposome · Asp8 peptide [47]	NR	Non-Asp8 loaded liposome; empty targeted liposome	Spine/femur fluorescence higher than non-Asp8 at 72 h (qualitative); Asp8 bone-affinity delivery	P1NP 9.53 ± 0.81 vs. 7.84 ± 0.38 ng/mL (non-Asp8); BFR 3.3 vs. 1.6 µm^3^/µm^2^/day; BV/TV +94.8% vs. OVX (*p* < 0.01)	Quantitative; incremental Asp8-associated effect supported by matched control
Curcumin/HPG–ALN prodrug · ALN [48]	NR	Non-ALN analog (HPGC); free Cur + free ALN	HAp binding 56.9% vs. 22.4% (non-ALN); pH release 78.3% (pH 5.4) vs. 28.7% (pH 7.4)	BV/TV ↑, N.Oc/BS ↓ vs. free Cur and free ALN (significant; values not tabulated in source)	Semi-quantitative; matched control at delivery level
Icaritin/ALN micelle · ALN [49]	NR	Non-ALN loaded micelle (PM-ICT); free ICT	HAp binding ~80% vs. ~10% (non-ALN); t½ 40.1/34.8/27.7 h (targeted/non-ALN/free); cortical pore 12.8 vs. 19.6 µm	BMD, BV/TV, stiffness higher than non-ALN micelle (*p* < 0.05–0.01); Ca/P ~1.68 (near sham)	Semi-quantitative; matched control
Curcumin/osteoblast-like membrane-coated PLGA NP · cell membrane (CXCR4/TNFR) [40]	NR	Uncoated Cur@NPs	Osteoblast uptake +87.4% vs. uncoated; macrophage uptake 59.2% vs. 91.0% (immune evasion)	BMD, Tb.BV/TV, Tb.N higher than uncoated (significant; values not tabulated)	Semi-quantitative; coated-vs-uncoated matched control (biomimetic interface)
Curcumin/ZIF-8 · Asp8 peptide, pH-responsive [27]	NR	Non-Asp8 (Cur@ZIF); free Cur	pH release ~90% (pH 5.5) vs. ~30% (pH 7.5); bone fluorescence higher, liver/kidney lower than non-Asp8 (qualitative)	PINP/BALP ↑, β-CTX/TRAP-5b ↓ vs. non-Asp8 (*p* < 0.001; values not tabulated)	Semi-quantitative; matched control (integrated targeting + responsive release)
Icariin/TC–DSPE-PEG liposome · tetracycline + PEG [26]	157.5 nm; EE 92.85%; DL 10.48%	Non-targeted PEG liposome; free drug	Highest HAp binding and longest bone-fluorescence retention (qualitative); PK	BMD, BV/TV, Tb.N higher than non-targeted (*p* < 0.05; values not tabulated)	Semi-quantitative; matched control (tetracycline + PEG interface)
Group B—Surface/interface module present but only free-drug or no matched carrier control (incremental surface effect not isolated)					
Astragaloside IV/ALN-mPEG-PLGA micelle · ALN [50]	45.3 nm; EE 96.16%	Free AS (no non-ALN carrier in vivo)	Bone-tissue conc. 112.8 ± 15.4 vs. 1.95 ± 0.17 µg/mL; relative BA 233.9%; HAp ~81% vs. 21% (blank)	BV/TV, BMD ↑ and CTX-1, IL-6 (36.4 vs. 53.9 pg/mL) ↓ vs. free AS (*p* < 0.01)	Semi-quantitative; module present but no matched control (strong exposure data)
Myricetin/ALN micelle · ALN [51]	NR	Free myricetin (no non-ALN carrier)	Relative BA 3.54× (AUC 55.5 vs. 15.7 h·µg/mL); HAp >80% vs. <20% at 1 h	BV/TV, BMD, Tb.N ↑ vs. free MY (significant; values not tabulated)	Semi-quantitative; module present but no matched control
Geniposidic acid/SDSSD–GPA conjugate · SDSSD peptide [52]	NR	Free GPA	Bone radiant efficiency ~4× liver (self-distribution; no free-drug distribution control); osteoblast enrichment	BMD, BV/TV, P1NP, MAR ↑ vs. OVX (bar-chart; values not tabulated)	Semi-quantitative; SDSSD module present but no matched control
Apigenin/E7-modified liposome · E7 peptide [53]	NR	Free drug	Fluorescence imaging; bone-associated enrichment	Greater BMD improvement than free drug	Qualitative; E7 module present but only free-drug comparison
Curcumin/ALN acid-responsive polyphosphazene · ALN + acid-responsive [54]	181.9 nm; DL 25.8%	NR	HAp binding; in vivo bone-associated delivery; acid-responsive release	Reduced bone loss	Semi-quantitative; integrated mineral-affinity/responsive design; no matched control
Celastrol/bone-affinity mesoporous silica · bone-affinity carrier [55]	NR	NR	High bone affinity; sustained release	Increased BMD and preserved trabeculae	Qualitative; bone-affinity design; no matched control
Daidzein/HAp nanoparticle · intrinsic mineral affinity [56]	NR	Free drug/model controls	Biomineralization; cellular uptake; PK/PD evaluation	Improved bone architecture and turnover markers	Semi-quantitative; HAp intrinsic mineral affinity; no matched non-HAp carrier
Group C—No dedicated targeting module; nanonization, self-assembly, or long circulation only (formulation-level benefit)					
Quercetin/SLN · none [57]	172.9 nm	Free quercetin	AUC 31,842 vs. 8951 ng·h/mL (3.5×); t½ 46.7 vs. 14.3 h; no bone-directed interface	Tibia BV/TV 13.0 ± 0.6 vs. 10.3 ± 0.7%; femur stiffness 245.8 vs. 198.9 N/mm; urinary CTX ↓ 36% vs. OVX	Quantitative (full tables); formulation-level (nanonization) benefit only
Naringenin nanosuspension · none [58]	NR	Free NRG; raloxifene; blank-NS	No bone-directed readout reported	Bone density 1.40 ± 0.29 vs. 0.69 ± 0.07 (free), 0.54 (OVX) g/cm^3^; serum OCN 3.89 vs. 2.47 ng/mL	Quantitative; formulation-level benefit only
Berberine + chlorogenic acid/carrier-free supramolecular nanoagonist · none (passive) [59]	DL 100%	Free BER; free CGA	t½ 13.3 vs. 4.2 h; AUC 5.9–10× free components; passive bone-associated enrichment after prolonged circulation	BV/TV 6.2%, Tb.N 1.20 mm^−1^, Tb.Sp 0.315 vs. 0.382 mm vs. OVX (*p* < 0.01)	Semi-quantitative; self-assembly/formulation benefit (passive)
Quercetin/phosphorylated carrier-free nanostructure · none [25]	NR	Free quercetin	Improved dispersibility, bioavailability, and bone affinity	Reduced trabecular bone loss	Semi-quantitative; integrated molecular-engineering benefit
Puerarin/TPGS long-circulating liposome · none (long circulation) [22]	76.63 nm; EE 95.08%; DL 7.84%	Free drug	In vivo imaging; PK; sustained release; no bone-directed interface	Improved bone phenotype	Formulation-level (prolonged circulation/systemic exposure) benefit
Nobiletin/PEG-PCL micelle · none [60]	~124 nm; EE 76.34%; DL 7.60%	Model/free-drug controls	Sustained release; no dedicated skeletal-targeting evidence	Reduced bone loss	Formulation-level micellar benefit

Note: EE, encapsulation efficiency; DL, drug loading; NR, not reported; BA, bioavailability; PK, pharmacokinetics; t½, terminal half-life; BFR, bone-formation rate; MAR, mineral apposition rate. Reported values are descriptive, not pooled; cross-study heterogeneity in cargo, carrier, model, dosing regimen and endpoints precludes formal meta-analysis. Comparisons with the free drug primarily reflect benefits arising from nanonization or the overall formulation design, whereas comparisons with a compositionally comparable carrier lacking the specific surface/interfacial module (Group A) provide a more direct basis for assessing incremental surface-engineering effects. HAp binding primarily indicates in vitro mineral affinity, while fluorescence/NIR imaging reflects carrier- or probe-associated distribution; bone-tissue drug concentration and validated quantitative exposure measurements provide more direct support for cargo exposure. “Values not tabulated” indicates the original study reported the comparison only as bar charts or significance levels.

**Table 3:** Methods commonly used to evaluate surface-engineered phytochemical nanomedicines in OVX/PMOP models.

Aspect	Typical Methods/Readouts	What It Mainly Reflects
In vivo distribution and bone-associated enrichment	IVIS; DiR/FITC/NIR imaging; time-series fluorescence; ex vivo organ imaging [89,90]	Carrier- or probe-level in vivo distribution and bone-associated enrichment; signal reflects the carrier or probe, not cargo concentration, and is best read with ex vivo imaging and matched non-targeted controls
Spatial localization within bone and marrow microenvironments	Bone-section confocal imaging with TRAP, CTSK, MMP-9, ALP, OCN, RUNX2, Col1, or BMSC/senescence markers [68,87,91,92]	Tissue-level spatial relationship between carrier signal and resorption or osteogenic regions; co-localization does not by itself demonstrate intracellular cargo exposure
Bone structure	micro-CT: BMD, BV/TV, Tb.N, Tb.Th, Tb.Sp, cortical parameters [93,94]	Post-treatment bone mass and microarchitecture (therapeutic outcome, not delivery)
Bone turnover	CTX-I, TRAP/TRAP-5b, PINP/P1NP, OC, BALP, RANKL/OPG [29,34,94]	Resorption/formation balance; interpreted with OVX timing and dosing window
Histology	H&E, TRAP, Masson/Goldner, calcein double labeling [95]	Trabecular continuity, osteoclast burden, matrix formation, dynamic mineralization
Quantitative cargo exposure	LC-MS/MS bone-tissue concentration; tissue-specific PK; radiotracing/PET/SPECT of the labeled species	Analyte-specific cargo exposure (LC-MS/MS) or quantitative biodistribution of the labeled species; interpretation depends on what is labeled and label stability
Systemic tolerability and major-organ safety	Body weight, hematology, hepatic/renal function, serum biochemistry, major-organ H&E [96,97]	Short-term systemic tolerability only; long-term and quantitative extraskeletal accumulation belong to the translational stage (Table 4)

**Table 4:** Translational considerations for surface-engineered phytochemical nanomedicines.

Consideration	What Mainly Matters	Current Status and Outlook
Basic characterization, serum stability, and batch consistency	Particle size, PDI, ζ potential, encapsulation efficiency, drug loading, release profile, batch-to-batch consistency	Characterization is relatively mature; batch consistency is the main weak link between formulation data and in vivo results
Surface engineering and bone-affinity testing	Ligand type and density, conjugation method, serum stability; HAp/bone-slice binding, serum competition, desorption	Bone affinity reflects ligand density and conjugation rather than ligand presence alone; differing assay conditions limit cross-study comparison, so binding data are best read together with in vivo distribution
Model relevance	Animal age, OVX duration, treatment onset, skeletal site, baseline bone mass	Determines how far OVX results transfer to human PMOP; aged and delayed-treatment OVX models improve clinical relevance
Long-term and cargo-specific safety	Repeated-dose tolerability, quantitative organ and extraskeletal accumulation, clearance kinetics; cargo-specific endocrine endpoints for phytoestrogen-like compounds	Most current data are short-term (Table 3); long-term, quantitative extraosseous accumulation and cargo-specific safety remain the main translational-stage gaps
From qualitative imaging to quantitative exposure	Separating carrier signal, cargo exposure, and therapeutic response using LC-MS/MS, radiotracing, PET/SPECT	Evaluation is shifting from qualitative imaging toward analyte-specific quantification, a main direction of the field
Multitarget phytochemical delivery as an adjunctive strategy	Relation to bisphosphonate–drug conjugates, bone-targeted nucleic-acid systems, and anti-RANKL/anti-sclerostin biologics	Adjacent strategies offer single, well-defined targets (with clinical precedent for biologics); the phytochemical route trades target precision for multitarget, natural cargoes, supporting an adjunctive rather than replacement role

Note: This table summarizes the discussion in Section 5; supporting references are cited in the corresponding text.

## Data Availability

No new data were created or analyzed in this study. Data sharing is not applicable to this article.

## Abbreviations

The following abbreviations are used in this manuscript:

ACC	amorphous calcium carbonate
ADME	absorption, distribution, metabolism, and excretion
ALN	alendronate
ALP	alkaline phosphatase
Asp8	octa-aspartic acid peptide
BA	bioavailability
BALP	bone-specific alkaline phosphatase
BMD	bone mineral density
BMP	bone morphogenetic protein
BMSC	bone marrow mesenchymal stem cell
BMSCs	bone marrow mesenchymal stem cells
BV/TV	bone volume/tissue volume
COL1/Col1	type I collagen
Cur@ZIF@Asp8	curcumin-loaded Asp8-modified zeolitic imidazolate framework-8 nanoplatform
CTX-I	type I collagen C-terminal telopeptide
CTSK	cathepsin K
CXCR4	C-X-C chemokine receptor type 4
DiR	1,1′-dioctadecyl-3,3,3′,3′-tetramethylindotricarbocyanine iodide
DL	drug loading
DSPE-PEG	1,2-distearoyl-sn-glycero-3-phosphoethanolamine-poly(ethylene glycol)
(DSS)6	six repeats of the Asp-Ser-Ser motif
EDO	estrogen-deficiency osteoporosis
EE	encapsulation efficiency
ER	estrogen receptor
FITC	fluorescein isothiocyanate
FXR	farnesoid X receptor
Glu6	hexa-glutamic acid
H&E	hematoxylin and eosin
HA/HAp	hydroxyapatite
HO-1	heme oxygenase-1
IVIS	in vivo imaging system
LC-MS/MS	liquid chromatography-tandem mass spectrometry
MAPK	mitogen-activated protein kinase
micro-CT	micro-computed tomography
MMP-9	matrix metalloproteinase-9
MOF	metal–organic framework
mPEG-PLGA	methoxy poly(ethylene glycol)-poly(lactic-co-glycolic acid)
NF-κB	nuclear factor kappa B
NFATc1	nuclear factor of activated T cells cytoplasmic 1
NIR	near-infrared
NIR-II	second near-infrared window
NLC	nanostructured lipid carrier
NP	nanoparticle
NPs	nanoparticles
NR	not reported
Nrf2	nuclear factor erythroid 2-related factor 2
OC/OCN	osteocalcin
OM/Cur@NPs	osteoblast-like cell membrane-coated curcumin-loaded nanoparticles
OPG	osteoprotegerin
OVX	ovariectomized/ovariectomy
PDI	polydispersity index
PEG	poly(ethylene glycol)
PEG-PCL	poly(ethylene glycol)-poly(ε-caprolactone)
PET	positron emission tomography
PINP/P1NP	procollagen type I N-terminal propeptide
PK	pharmacokinetics
PLGA	poly(lactic-co-glycolic acid)
PMOP	postmenopausal osteoporosis
PRISMA	Preferred Reporting Items for Systematic Reviews and Meta-Analyses
RANKL	receptor activator of nuclear factor-κB ligand
ROS	reactive oxygen species
RUNX2	runt-related transcription factor 2
SASP	senescence-associated secretory phenotype
SDSSD	Ser-Asp-Ser-Ser-Asp peptide
SGPA	SDSSD–geniposidic acid system
siRNA	small interfering RNA
SIRT1	sirtuin 1
SLN	solid lipid nanoparticle
SPECT	single-photon emission computed tomography
Tb.N	trabecular number
Tb.Sp	trabecular separation
Tb.Th	trabecular thickness
TC	tetracycline
TNF-α	tumor necrosis factor alpha
TPGS	D-α-tocopheryl polyethylene glycol 1000 succinate
TRAF6	TNF receptor-associated factor 6
TRAP	tartrate-resistant acid phosphatase
ZIF-8	zeolitic imidazolate framework-8
